# Tumour dormancy and clinical implications in breast cancer

**DOI:** 10.3332/ecancer.2013.320

**Published:** 2013-05-21

**Authors:** L Gelao, C Criscitiello, L Fumagalli, M Locatelli, S Manunta, A Esposito, I Minchella, A Goldhirsch, G Curigliano

**Affiliations:** Early Drug Development for Innovative Therapy Division, European Institute of Oncology, Milan, Italy

**Keywords:** tumor dormancy, breast cancers, angiogenesis, microenvironment, immune surveillance

## Abstract

The aim of adjuvant therapy in breast cancer is to reduce the risk of recurrence. Some patients develop metastases many years after apparently successful treatment of their primary cancer. Tumour dormancy may explain the long time between initial diagnosis and treatment of cancer, and occurrence of relapse. The regulation of the switch from clinical dormancy to cancer regrowth in locoregional and distant sites is poorly understood. In this review, we report some data supporting the existence of various factors that may explain cancer dormancy including genetic and epigenetic changes, angiogenic switch, microenvironment, and immunosurveillance. A better definition and understanding of these factors should allow the identification of patients at high risk of relapse and to develop new therapeutic strategies in order to improve prognosis.

## Introduction

The term ‘dormancy’ was introduced in the first half of the 20th century by the Australian pathologist Rubert A. Willis in [1]. Tumour dormancy is part of the process of tumour progression characterized by the presence in specific organs of tumour cells that do not increase the tumour burden. Clinically, it is characterised by a long disease-free interval between primary tumour and relapse. The prevalence of clinical dormancy, which is frequently observed in several solid tumours such as breast, renal, thyroid, prostate cancer and melanoma [2], is still unknown. However, in recent years, molecular and genetic characterisation of disseminated tumour cells (DTCs) and circulating tumour cells (CTCs) has helped us to better understand the mechanisms underlying tumour dormancy [3]. Breast cancer (BC) remains the most frequently diagnosed cancer in women. Despite major advances in BC treatment, mechanisms of resistance and tumour heterogeneity may still render standard therapies ineffective in killing some subsets of cancer cells, such as dormant cells, thus resulting in late relapse. The biology of BC recurrence should be reviewed in order to improve the prognosis and therapies of these patients. The mechanisms underlying the switch from clinical dormancy to cancer regrowth are poorly understood. In this review, we will discuss biological mechanisms and possible clinical implications of tumour dormancy in BC patients.

## Mechanisms underlying tumour dormancy

**Figure 1:**
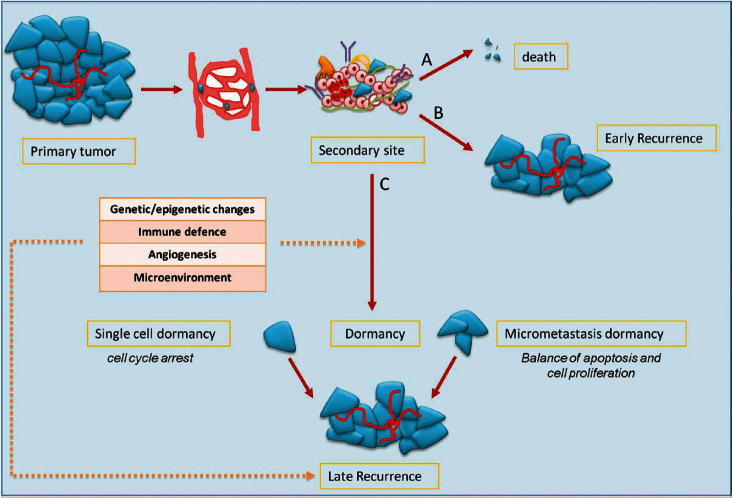
the fate of tumour cells in metastatic process and dormancy: tumour cells released by primary tumour can die, grow, or enter into a dormant phase. After a variable period, even decades, cells can exit from dormancy causing later relapse.

Mechanisms of metastasis include the release of cancer cells from primary tumour; invasion of the surrounding stromal tissue, lymphatic, and blood vessels as CTCs; and then colonisation of target organs as DTCs. The fate of DTCs may have several scenarios: (1) spontaneous apoptosis or cell killing by recognition of immune effectors; (2) cell proliferation with clinical early relapse; and (3) state of tumour dormancy (Figure 1).

Preclinical data have shown that tumour dormancy can occur both as a single dormant cell and as micrometastases. Single dormant cells are defined as cells undergoing cell cycle arrest and have the ability to develop mechanisms to evade immune surveillance [4, 5]. Only a small number of dormant cells (about 2%) can initiate growth as micrometastases, and an even smaller number (about 0.02%) grow into macroscopic tumours [6]. In the micrometastasis dormancy, there is a state of balance between apoptosis and cell proliferation resulting in no increase of tumour burden [7].

For reasons that are still not clear, after variable periods, even years after the diagnosis of primary tumour, dormancy ends and cancer cells start to proliferate causing late relapse. There are many factors that might explain the regrowth of cancer cells, hence regulating the entry in or exit from tumour dormancy: (1) genetic and epigenetic changes; (2) angiogenic switch; (3) immune system and immune evasion; and (4) microenvironment.

## Genetic and epigenetic changes

Genetic alterations have been observed in bone marrow cancer cells of BC patients. DTCs in the early setting do not present many of the end-stage genetic aberrations that are frequently observed in the metastatic setting. Data from Klein *et al *[8] demonstrated that tumour cells in patients with different metastatic disease had several aberrations at a genomic level and had a homogeneous profile. Dormant DTCs from patients with non-metastatic disease were genetically extremely heterogeneous, and their chromosomal abnormalities are very different from their matched primary tumours [9]. It is possible that DTCs originate from earlier stages of cancer progression and, over a variable period, only some of them accumulate genetic changes required to metastasise. The chromosomal heterogeneity among DTCs of an individual patient and the clonal expansion after clinically evident metastases suggests that chromosomal aberrations might be important for the outgrowth of DTCs and may be for systemic spread [10]. To date, no specific genetic signature has been identified that could explain the molecular mechanisms associated with tumour dormancy, but several studies suggest genes and molecular pathways that might govern dormancy and escape from dormancy.

Xenograft models of human tumour dormancy from four tumour types (human breast carcinoma, glioblastoma, osteosarcoma, and liposarcoma) were studied [11]. Genome-wide transcriptional analysis was used to compare gene expression profiles of cells that form either dormant or fast-growing tumours for each of the four tumour types. Genes that were differentially regulated between dormant and fast-growing tumours had the same expression pattern in all tumour types. A consensus gene signature distinguishing all four dormant versus rapidly proliferating tumours was generated. Angiogenesis was the most differentially regulated pathway between the dormant and the fast-growing tumour phenotypes. In fact, the switch of dormant tumours was associated with down regulation of angiogenesis inhibitor thrombospondin (TSP) and decreased sensitivity of angiogenic tumours to angiostatin.

In many other studies, other pathways (such as endothelial cell-specific molecule-1, 5′-ecto-nucleotidase, tissue inhibitor of metalloproteinase-3, epidermal growth factor receptor (EGFR), insulin-like growth factor receptor, and phosphatidylinositol 3-kinase signalling) and genes (metastatic-suppressor genes, that is, KISS1 metastasis-suppressor, CD82, NME/NM23 nucleoside diphosphate kinase 1) [12] have been described as implicated in tumour dormancy process.

In recent years, particular interest has been attributed to microRNAs (miRNAs) small non-coding RNA molecules, considered as regulators of gene expression. A recent review identified a set of 19 miRNAs that control the phenotypic switch of human dormant breast carcinoma, glioblastoma, osteosarcoma, and liposarcoma tumours to exponential growth [13]. High expression levels of 16 miRNAs were found in dormant tumours. Downregulation of these miRNAs correlated with the switch of dormant tumour to the fast-growing angiogenic tumour. The expression pattern of two dormancy associated miRNA (DmiRs) (miR-580 and 190) correlated with disease stage in human glioma specimens and reconstitution of a single DmiR (miR-580, 588 or 190) led to phenotypic reversal of rapidly proliferating angiogenic tumours towards prolonged tumour dormancy.

In addition to genetic changes, epigenetic alterations are frequent both in dormancy and proliferation mechanisms. Metge *et al *[14] demonstrated that in 20 BC samples, 45% of the primary tumours and 60% of the matched lymph node metastases displayed hypermethylation of breast cancer metastasis suppressor 1 (BRMS1) promoter, and this aberrant methylation caused loss of its expression. This and other epigenetic changes can occur in dormancy, and future studies will aim at understanding these mechanisms. In conclusion, molecular changes at genetic and epigenetic levels might play a central role in the regulation of dormancy, and further studies will likely validate some of these pathways or genes, leading to the development of therapeutic strategies addressing the induction and/or maintenance of a dormant state.

## Angiogenesis

**Figure 2:**
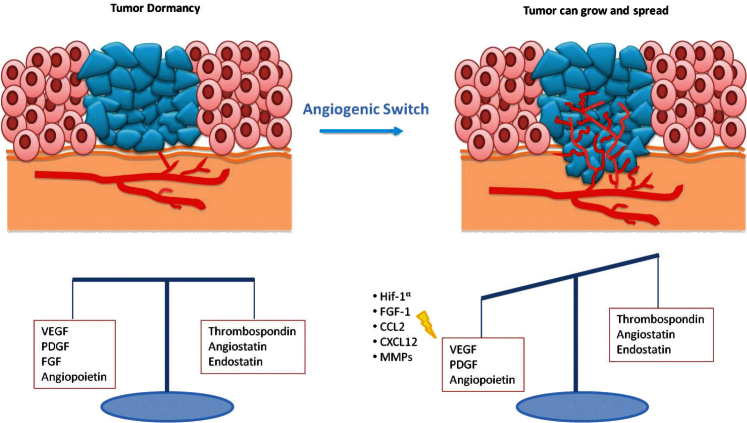
In dormant micrometastasis, there is a balance between angiogenic and anti-angiogenic factors. When this equilibrium is destroyed by the prevalence of angiogenic factors, tumour can grow.

Expansion of a tumour mass requires vascular supply by angiogenesis [15]. Some studies showed that dormant micrometastases are avascular tumour in which there is a balance between proangiogenic factors (vascular endothelial growth factor (VEGF), PDGR, fibroblast growth factor (FGF), angiopoietin) and anti-angiogenic factors (endostatin, agiostatin, TSP), with a slight prevalence of the latter ones (Figure 2) [16]. The capacity of micrometastasis to grow mainly depends on their ability to secrete angiogenic factors and downregulate angiogenic suppressors.

Naumov *et al *[17] developed a model of human tumour dormancy that compares non-angiogenic and angiogenic cancers, by using cell lines of BC, osteosarcoma, and glioblastoma. When non-angiogenic human cancer cell lines were subcutaneously implanted in SCID (severe combined innumodeficient) mice, most of the resulting tumours remained microscopic (<1-mm diameter) for prolonged periods. Some of them became angiogenic and were used to isolate angiogenic tumour cells. Unlike non-angiogenic cell lines, angiogenic cancer cells induced palpable tumours more frequently and earlier. Considering that no significant differences emerge by comparing the proliferation and apoptosis rates between the two cell types, authors concluded that the prolonged dormancy in the non-angiogenic cell lines was likely due to decreased angiogenic capacity, rather than the mechanisms of quiescence or apoptosis. This conclusion was supported by genome-wide transcriptional analysis that showed significant differences between different cancer cells of genes associated with angiogenesis [11]. The ability of a tumour to progress from a non-angiogenic to an angiogenic phenotype is termed the ‘angiogenic switch’. This switch is driven by increased expression of angiogenic proteins and decreased expression of angiogenesis inhibitors by tumour cells and by stromal cells [18].

In several studies, investigators developed different models to show the importance of angiogenesis in the escape from dormancy and to induce tumour growth and progression [19]. In these studies, dormancy was induced by the presence of potent inhibitors of angiogenesis [20]. Moreover, the addition of angiogenic factors resulted in tumour cells escaping from tumour dormancy and switching to a rapidly growing state.

Giuriato *et al *[21] demonstrated that inactivation of MYC led to regression of haematopoietic tumours in transgenic mice, except in tumours that lost p53 function. Histological examination revealed that upon MYC inactivation, the loss of p53 led to a deficiency in TSP-1 expression and the subsequent inability to shut off angiogenesis. Restoration of p53 expression in these tumours re-established TSP-1 expression.

This allowed the suppression of angiogenesis and subsequent sustained tumour regression upon MYC inactivation. Cancer cells undergoing angiogenic switch also produce chemoattractants and mitogens, including HIF1-α (hypoxia-inducible factor 1-alpha), VEGF, FGF-1, CCL2 (C-C motif chemokine 2), and CXCL12 (stromal cell-derived factor 1), for endothelial cells and proangiogenic immune cells [22, 23]. Such cells also secrete matrix metalloproteinases (MMPs) that free angiogenic factors from the surrounding stroma and remodel the extracellular matrix to blood vessel branching [24]. Heat shock protein 27 kDa (HSP27) has finally been shown to be important in the study of dormancy and in the angiogenic switch, promoting the secretion of proangiogenic factors (VEGF-A, VEGF-C and b-FGF). Straume *et al *[25] demonstrated that stable downregulation of HSP27 in angiogenic human BC cells followed by long-term tumour dormancy *in vivo*. Conversely, overexpression of HSP27 in non-angiogenic cells resulted in expansive tumour growth *in vivo*.

Several efforts are needed to better understand the angiogenesis switch and, accordingly, identify potential therapeutic targets to either induce or maintain tumour dormancy or, conversely, to induce dormant cell death.

## Immune surveillance

**Figure 3:**
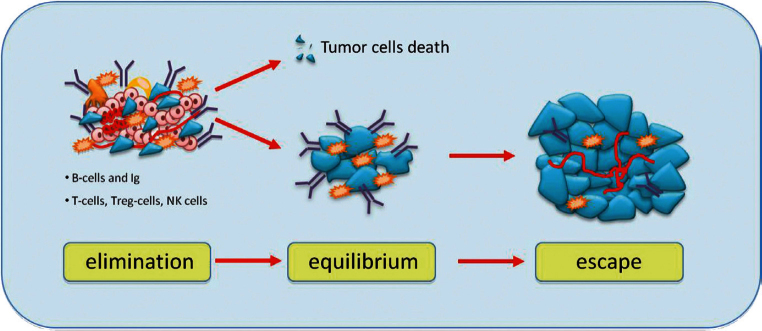
Immunoediting hypothesis: immunity coordinated by T cells and B cells causes elimination of tumour cells or some of them enter a phase to equilibrium and tumour border does not increase. After a variable period, cells can escape from immunosurveillance and grow, causing tumour mass expansion.

Although the role of the immune system in dormancy has been studied for a long time, it is not yet well established. It is possible that a balance between immune response modulated by T cells effectors and tumour cells leads to a long-term tumour dormancy. According to the cancer immune-editing hypothesis, tumour development goes through three phases: elimination, equilibrium and escape (Figure 3) [26]. The elimination phase represents the cancer surveillance, in which cells and molecules of the innate and adaptive immune systems may eradicate the tumour and protect the host against tumour development. However, if this process is not successful, the tumour cells may enter an equilibrium phase in which they may either be maintained chronically or immunologically sculpted by immuno-‘editors’ to produce new populations of tumour variants. The equilibrium phase corresponds to dormancy. The duration of this equilibrium depends on various factors, such as the chromosomal instability of the cancer cells. Tumour cells accumulate chromosomal aberrations and, over time, genetic instability translating in another phenotype enables them to avoid the antitumour immune response and to escape dormancy [27]. Several studies demonstrate that both the humoral and the cellular immune system contribute to maintain the state of dormancy. It is possible to induce tumour dormancy in immune-competent hosts by prior immunisation against tumour cells. For example, in the BCL1 mouse lymphoma model, tumour dormancy can be induced by immunisation with BCL1-derived immunoglobulin (Ig) to generate an anti-idiotype immune response. This usually induces cell cycle arrest, and about 70% of mice maintain tumour cells in the spleen [28]. Alterations in the tumour cells seemed to be responsible for loss of anti-Id sensitivity and the subsequent escape from dormancy.

The cellular immune response plays a critical role for maintaining cancer dormancy. This is supported by the evidence that depletion of CD4+ and CD8+ T cells in mouse models causes escape of tumour cells from dormant state [29]. In humans, when patients with BC and healthy women were compared, the proportion of CD4+ and CD8 memory T cells was higher in bone marrow of patients than in healthy women [30]. Tumour cells can circumvent immune response through overexpression of B7 homolog 1 (B7-H1) that inhibit T-cell activation and the cytotoxic T lymphocyte (CTL) response [31]. Moreover, several studies demonstrated that DTCs became resistant to T-cell-mediated lysis and had less ability to CTL to secrete interferon (IFN)-g and tumour necrosis factor (TNF)-a [32].

Finally, several immune cells are present in the tumour microenvironment, such as regulatory T-cells (TREG cells) [33] and tumour-associated macrophages (TAMs) [34] that secrete mitogens, proangiogenic factors, MMPs and cytokines, might allow the escape from dormancy thus stimulating the tumour growth.

A better understanding of the influence of the immune system on dormancy could lead to the development of immunological therapies in order to prevent the progression of the tumour and then the switch from the state of dormancy to tumour growth.

## Microenvironment

The fate of solitary DTCs is influenced by interactions between tumour and host occurring in primary tumours and target organs. Studies *in vivo *demonstrate that a loss of growth signals and cell-to-cell signalling attachments can lead to dormancy, suggesting that a microenvironment, with inappropriate cell contacts and signalling, can contribute to tumour cell dormancy [35]. Conversely, tumour cells able to establish appropriate interactions in their new environment can grow and form successful metastases. Bragado *et al *[36] described three potential scenarios that might explain the interaction of tumour cells and microenvironment.

In the first scenario, authors suggest that target organ microenvironment determines the fate of DTCs. If DTCs seed in a permissive microenvironment, DTCs proliferation will be promoted. Instead, if DTCs seed in an unfavorable microenvironment, there will be inactivation of proliferative signals or interaction with growth-restrictive signals that in turn will lead to dormancy.

The second scenario proposes that reciprocal influence of the primary tumour and ‘microenvironment stress’ induced by hypoxia or therapies in primary sites generates signatures that can inform about DTCs survival, dormancy, or proliferation. Published data prove that gene signatures present in the primary tumours predict long-term relapse in the absence of the primary tumour from which the signature derived [37]. The dormancy signature identified in dormant D-HEp3 cells predicted for longer metastasis-free periods in oestrogen receptor (ER) positive BC primary tumours. In contrast, when this signature was under-represented, recurrences were more frequent. This suggested that while the signature does not influence primary tumour growth, it might induce slower progression probably through dormancy program.

In the third scenario, authors hypothesised that ‘pre-malignant’ cells can undergo epithelial mesenchymal transition (EMT), making them invasive and promoting early dissemination. But it is possible that these early DTCs, carrying specific genetic and epigenetic alterations, are not able to initiate metastatic growth and thus undergo dormancy. During the earliest stages of progression, the EMT is reversible; moreover, the stress signalling or suppressive signals from the microenvironment are able to maintain DTCs quiescent till arrival to the target organ. However, subsequent genomic alterations eventually produce cells able to initiate metastasis.

**Figure 4:**
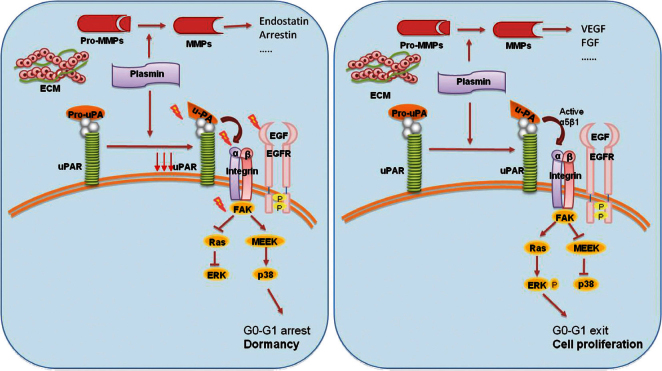
The activation of p38 pathway through the fibronectin receptor alpha 5b1 causes the cell cycle arrest and dormancy. The activation of ERK pathway instead leads to cell proliferation and tumour mass expansion. Furthermore, the secretion of MMPs by stromal cells determines the release of angiogenic or anti-angiogenic factors.

Experimental models suggested the involvement of the urokinase receptor (uPAR), extracellular signal-regulated kinase (ERK) and p38 pathways in the regulatation of dormancy (Figure 4) [38]. In fact, uPAR seems to play a central role to regulate the balance between tumour cells proliferation and tumour dormancy, it is also found to be expressed by DTCs, and its expression might potentially be considered a predictive marker for unfavourable prognosis [39]. uPAR interacts and actives the fibronectin receptor alpha5b1 integrin. This complex recruits focal adhesion kinase (FAK) and EGFR, which promotes mitogenic signals through the ERK pathway. In squamous carcinoma cells (HEp3), it was shown that reduced uPAR expression made these cells incapable of binding efficiently to fibronectin [35]. This resulted not only in reduced FAK and EGFR signalling but also in p38/MAPK activation. *In vitro *and *in vivo *studies showed that the downregulation or blocking of uPAR, beta1integrin, FAK or EGFR, alone or in combination causes activation of p38/MAPK pathway resulting in a cell cycle arrest and dormancy (Figure 4) [40, 41].

Thus, the proposed molecular mechanisms of the growth inhibition that occurs in dormancy involve either activation of the p38/MAPK pathways or inhibition of the ERK/MAPK pathways. Instead, the interaction of uPAR with fibronectin receptor and EGFR leads a shift from the state of dormancy to cell proliferation. The ratio of ERK to p38 is very important for dormancy; a high ERK to p38 ratio is linked to proliferation, whereas low ERK to p38 expression ratio is linked to growth arrest and dormancy [42].

In addition, *in vivo *studies demonstrated that ATF6 alpha promotes survival of dormant tumour cells through the upregulation of Rheb and activation of mTOR signalling, independent of Akt [43]. Finally, the microenvironment might contribute to tumour dormancy or its switch to metastatic growth because the secretions and expression of MMPs by leukocytes and macrophages can lead to the release of angiogenic factors (FGF and VEGF) [44] or anti-angiogenic factors (endostatin, restin, arrestin, the three chains of collagen IV, and macrophage elastase) [45].

## CTCs, DTCs, and cancer stem cell hypothesis

Several studies evaluated the prognostic value of CTCs or DTCs at the time of initial diagnosis and before the start and/or the end of adjuvant chemotherapy [46, 47]. DTCs and CTCs are theoretically the targets of adjuvant treatment and their fate after systemic therapy could be a potential useful marker for the estimation of the risk of recurrence.

In a recent study, authors suggested a possible value of CTCs in predicting the risk of late relapse, defined as relapse that occurs at least two years after the end of adjuvant chemotherapy in 312 patients with operable BC at stage I–II–III either hormonal receptor positive or negative, who were disease free during the first two years of follow-up [48]. The patients were divided into four groups: the first group included patients with CTCs persistently positive and 36.4% of whom experienced disease relapse; in the second group, patients without detectable CTCs were included and only 11.2% of whom showed disease relapse; the third group included patients with CTCs during the first two years. These patients had similar relapse risk to the persistently negative patients (7.8%). In the last group of patients with detectable CTCs after the first two years, the relapse risk was 18%. Patients with CTCs persistently positive had distant relapse-free survival (*P *= 0.001) and overall survival (OS) (*P *= 0.001) worse than other groups of patients, regardless of ER status. These results suggest that resistant dormant cells have a proliferative and survival advantage; they may resist the conventional anticancer therapy and may start to proliferate even many years after the diagnosis of primary tumour. Why not all patients with detectable CTCs develop late recurrence, while some patients without detectable CTCs relapse, could be explained by the evidence that CTCs showed significant genetic heterogeneity.

It was also investigated the potential role of DTCs in predicting risk of relapse after the completion of adjuvant chemotherapy. Six hundred and seventy-six patients with operable BC stage I–III were analysed [49]. Median follow-up time was 89 months from diagnosis. Persistent DTCs were detected in 15.5% of patients. Distant relapse-free survival and OS were significantly shorter in patients with DTCs compared with patients without DTCs (log-rank test: *P* = 0.002 and *P* < 0.001, respectively) during the first five years following cancer diagnosis.

The detection of these cells after therapy could be considered as an indirect evidence for the presence of dormant cells chemotherapy/ hormonal therapy resistant. However, not all patients with detectable DTCs or CTCs will experience late recurrence. Thus, additional prognostic markers are needed to define those patients are at high risk of late relapse.

In 2003, Clarke *et al *[50] first described cancer stem cells (CSCs) in a solid tumour. Several studies suggest that tumours include heterogeneous populations of CSCs and non-stem cancer cells. These two groups of cells interact with each other and their microenvironment. Non-stem cancer cells rapidly become the dominant population in a tumour and induce the CSCs into quiescence. Because of the limited proliferation capacity of non-stem cancer cells, the tumour population eventually ceases to expand, and the tumour enters and maintains the state of dormancy even decades until unknown events lead the CSCs to be reactivated to renewed proliferation in order to drive tumour progression beyond dormancy [51]. According to this theory, dormant tumour cells might represent CSCs. In fact, various evidences suggest that a subpopulation of cancer cells exhibits stem-like properties and is capable of tumour initiation, invasive growth, and late relapse [52]. These CSCs have the ability to self-renew to give rise to other stem-like cells, as well as undergo differentiation to give rise to the non-stem cancer cells that form the rest of the tumour. It has been proposed that, in some patients, the cancer cells remain dormant until some unknown event triggers renewed proliferation, or alternatively, it is possible that the DTCs arise from CSCs, and only when CSCs disseminate and subsequently self-renew, the patients will relapse with macroscopic metastases [53]. CSCs express high levels of anti-apoptotic proteins (such as members of the Bcl-2 family) [54] and can resist apoptotic proteins by a number of mechanisms, including activation of the Hedgehog (HH) pathway and dysregulated transforming growth factor-beta (TGF-beta) signalling [55].

Moreover, some studies investigated the interactions between the microenvironment and CSCs. These cells could find (or create) a new specialised microenvironment or ‘tumour niche’ in secondary sites that generates extrinsic factors that control stem cell number, growth, and differentiation. It could be hypothesised that CSCs remain dormant in their niche either as solitary cells or as dormant micrometastases until they are activated by improper signalling from the microenvironment [56]. Furthermore, despite a limitless self-renewal capacity, CSCs are relatively quiescent and divide infrequently unless activated. Since many cancer molecules are designed to kill actively dividing cells, CSCs may escape cytotoxic drugs, and this is important in disease relapse. For example, it has been shown that stem-like subpopulation of cancer cells expresses high levels of ATP-binding cassette (ABC) transporters that can actively efflux drugs and shield them from the adverse effects of chemotherapeutic insult [57]. Finally, a recent research suggests that CSCs can control EMT process, during which epithelial cells acquire the ability to invade, resist apoptosis and disseminate. The EMT may not only contribute to the self-renewal ability and drug resistance of these cells but may also be responsible for creating and maintaining CSCs [58].

## Clinical implications

Since early tumour detection is a critical determinant of survival in patients with cancer, the recognition of dormant tumours and/or cells and their possible eradication with targeted therapies is one of the major goals of care of BC survivors.

Currently, there are no markers able to exactly predict the risk of late recurrence, and it is not possible to predict which dormant tumours and/or cells will eventually grow and which will stay dormant and will never switch to the rapidly proliferating phenotype [59]. We do not know if any features tumour or patient related are able to predict, at diagnosis, which patients will develop late metastases after a period of dormancy.

In order to overcome this medical need, it might be useful to develop gene signature as a means by which tumour behavior can be predicted. Several genes involved in many cellular pathways have been identified and have been associated with the conversion of dormant lesions into fast-growing tumours, and they could represent potential targets and markers for tumour dormancy. For example, different cellular and animal models showed that both MYC and Ras significantly affected the dormancy, through the regulation of expression of genes involved in angiogenesis [60]. The identification and the modulation of activities of these and other genes might help us to avoid tumour progression. However, it is not clear if these genes and proteins directly contribute to control tumour dormancy or are just correlated with the disease state.

Kim *et al *[61] suggested a correlation between gene signature and the biological features of the tumour, such as hormonal receptors expression. The risk of relapse in the first 5 years after diagnosis is lower in the ER+ disease than ER− disease, but patients with hormone dependent disease have a greater chronic annual risk of relapse than those with ER− tumours [62]. The question arises as to whether these differences are related to different growth rate patterns or to differences in dormancy phenomenon. Based on expression profiles obtained from BC cells and clinical samples, the authors generated a gene signature for tumour dormancy, in which they considered genes upregulated and gene down-regulated in dormant cells and then they defined the dormancy score. They found that the dormancy scores were significantly higher in ER+ tumours compared with ER− tumours (*P *< 0.0000001) [61]. Moreover, they demonstrated that a higher dormancy score was significantly associated with higher metastasis-free proportion. So it would be useful to develop the exact dormancy signature for each BC subtype to define both the prognosis and the therapeutic possibilities.

The identification of miRNAs associated with tumour dormancy might contribute to understand tumour dormancy and manipulation of their expression level could help us to delay or prevent the dormancy periods [13].

In addition to CTCs, DTCs, and CSCs, various efforts are being made to identify biomarkers in circulation that correlate with the presence of dormant tumours, such as platelet-associated PF-4 [63]. Carlsson *et al *[64] identified a 21-protein signature from 240 sera of 64 patients with primary BC. They assessed the risk of developing distant relapse after the primary surgery for each patient, using his or her molecular portrait. This risk assessment was not dependent on the type of adjuvant therapy given to the patients.

The dormant phase of tumours is also a promising therapeutic target. Dormant tumour cells are refractory to current cancer therapies. In an experiment using mouse mammary carcinoma cell lines, treatment with doxorubicin reduced the size of large metastases but did not reduce the number of solitary dormant cells [65]. Although quiescent, dormant cells are difficult to kill, and it could be possible to destroy them after they have reinitiated growth. Appropriate targeted drugs should be developed in order to eliminate or control these persistent tumour cells and thereby prevent their occasional transformation into growing metastases. To meet these endpoints, we have to target mechanisms underlying dormancy such as genetic and epigenetic changes with treatments that might modulate activity of dormancy-associated genes or with DNA methyltransferase (DNMT) and histone deacetylase (HDAC) inhibitors that might constitute a promising strategy for therapy. It might be useful to develop therapeutic strategies to regulate balance between p38 and ERK pathways, for example, by blocking the uPAR, fibronectin receptor, but it would be complicated because of the limited specificity for cancer cells.

Angiogenesis is another potential therapeutic target. Many anti-angiogenic drugs are currently approved in the treatment of metastatic cancer, such as Bevacizumab. To evaluate their capacity to influence dormancy, these agents should be tested in patients at high risk for relapse, without evidence of disease. Results from recent trials (AVANT and NSABP-08) failed to show a clear benefit from the antiangiogenic therapy bevacizumab when it was given as one year of adjuvant treatment in early-stage colon cancer [66, 67]. In the USA, large intergroup trial examines the impact of postoperative Sunitinib (a small-molecule tyrosine kinase inhibitor that inhibits VEGFRs and PDGFRs, among other targets) versus Sorafenib (a small molecular inhibitor of several tyrosine protein kinases and Raf kinases) versus placebo in patients with kidney cancer at moderate-to-high risk for relapse after nephrectomy, and it has now completed accrual. The results of this adjuvant study will determine the efficacy of anti-angiogenic drugs in delaying the relapse of cancer.

Immunological therapy might represent a possible strategy to prevent late recurrence. Most of the vaccines are tested in patients with metastatic disease. Therapeutic vaccines may be more effective in patients with microscopic disease or in adjuvant setting at high risk or relapse, because this therapy could prevent the loss of immunological surveillance and promote persistent dormancy. A limited number of adjuvant trials are in progress.

Many dormant cancer cells metastasise to the bone marrow as blood flow is high in this area. The tumour cells also produce adhesive molecules that bind them to marrow stromal cells and bone matrix (osteo-niche). In 1889, Stephan Paget described for the first time this ‘seed-and-soil hypothesis’ of the mechanism of bone metastasis [68]. The reciprocal interaction between cancer cells and the bone microenvironment causes the production and the release of angiogenic factors, growth factors, and bone-resorbing factors that may activate DTCs from a dormant to a proliferative state resulting in tumour growth and bone destruction [69]. These factors also increase the expression of receptor activator of nuclear factor-*k*B ligand (RANKL), a potent inducer of osteoclast formation. RANKL binds its receptor, RANK, on the surface of osteoclast precursors and signals through the nuclear factor-*k*B (NF*k*B), and Jun N-terminal kinase (JNK) pathways induce the formation of osteoclasts and promote osteoclast survival. RANKL inhibitors (i.e. denosumab) block the activation, survival and differentiation of osteoclasts from their precursors resulting in complete absence of osteoclasts in the treated bone.

Traditionally, bone-targeted agents are used as a therapy to prevent or reduce the incidence of skeletal-related events in patients with malignant bone disease. However, recent evidences suggest that they may act as antitumour agents [70], able to delay disease progression and prolong survival in solid tumours such as BC [71]. In the AZURE trial, treatment with zoledronic acid did not show a statistically significant increase in DFS compared with standard therapy alone in the overall (intention to treat) population, but, among patients who were postmenopausal for at least five years before study entry, treatment with zoledronic acid demonstrated a statistically significant reduction of risk of DFS events by 25% and of the risk of death from any cause by 26% [72]. There are also ongoing adjuvant studies with denosumab; ABCSG-18 [73] is a placebo-controlled study of monthly administration of denosumab 60 mg for six months in postmenopausal women receiving an aromatase inhibitor, whereas the D-CARE study [74] is evaluating a more intensive schedule of denosumab 120 mg, administered initially monthly for six months and then every three months thereafter in stage II–III BC.

Finally, patients with ER+ tumours might derive benefit from extended endocrine therapy. As discussed above, the initiation of metastatic growth is not equivalent to the dissemination of tumour cells [75]. This suggests that a single treatment or a combination of therapies, introduced at varying time intervals, could be necessary to interrupt the process of clinical recurrence. Recent clinical trials in BC support the idea that continued therapy or therapy that is applied late in follow-up may be of benefit in preventing cancer recurrence [76]. Ma17 trial evidenced that the extension of endocrine therapy with an aromatase inhibitor, letrozole, for five years after the initial five years of tamoxifen treatment resulted in an important reduction in the risk of relapse and in an improvement in disease-free survival [77]. SOLE trial, a phase III ongoing trial, will evaluate whether continuous letrozole *versus *intermittent letrozole treatment after four to six years of adjuvant endocrine therapy, can reduce the risk of relapse in early-stage BC patients. These clinical trials in hormone-dependent BC suggest that dormant cells remain vulnerable to this therapy. The open question is to evaluate the risk and benefit of long-term therapy.

## Conclusion

A better understanding of the regulatory mechanisms that govern the state of dormancy will help us to identify markers of early tumour progression and to detect early tumour cells prior to their rapid growth in order to treat tumours or recurrent cancer years before they become symptomatic. This could lead to a reduction in cancer mortality. Moreover, future studies on tumour dormancy could lead to the development of novel targeted strategies for eliminating dormant tumour cells or for maintaining dormant status of tumour cells, keeping the disease in a chronic state.
